# Partial Stent-in-Stent Method with an Uncovered Self-Expandable Metallic Stent for Unresectable Malignant Hilar Bile Duct Obstruction

**DOI:** 10.3390/jcm13030820

**Published:** 2024-01-31

**Authors:** Takuya Shimosaka, Yohei Takeda, Taro Yamashita, Yuta Seki, Shiho Kawahara, Takayuki Hirai, Noriyuki Suto, Yuri Sakamoto, Wataru Hamamoto, Hiroki Koda, Takumi Onoyama, Kazuya Matsumoto, Kazuo Yashima, Hajime Isomoto, Naoyuki Yamaguchi

**Affiliations:** 1 Division of Gastroenterology and Nephrology, Department of Multidisciplinary Internal Medicine, Faculty of Medicine, Tottori University, Nishi-cho 36-1, Yonago 683-8504, Japan; shimosaka.271@gmail.com (T.S.); yama_t11@yahoo.co.jp (T.Y.); leptnater@gmail.com (Y.S.); kawahara.hp1@gmail.com (S.K.); t.hirai@tottori-u.ac.jp (T.H.); d22m1004u@edu.tottori-u.ac.jp (N.S.); yuri.sakamoto@me.com (Y.S.); hamamoto_trr@yahoo.co.jp (W.H.); po.polnga.3.negaiwo.xxx@gmail.com (H.K.); golf4to@yahoo.co.jp (T.O.); matsumotokazuya@tottori-u.ac.jp (K.M.); yashima@tottori-u.ac.jp (K.Y.); isomoto@tottori-u.ac.jp (H.I.); 2 Department of Endoscopy, Nagasaki University Hospital, Sakamoto 1-7-1, Nagasaki 852-8501, Japan; naoyuki3334@hotmail.com

**Keywords:** unresectable malignant hilar bile duct obstruction, biliary stent, metallic stent

## Abstract

(1) **Background:** There is controversy regarding stent placement for unresectable malignant hilar biliary obstruction (UMHBO). We mainly use the partial stent-in-stent (PSIS) method with an uncovered self-expandable metallic stent (UCSEMS) based on the drainage area and patency period. In this study, we investigated the usefulness and safety of the PSIS method. (2) **Methods:** In total, 59 patients who underwent the PSIS method for UMHBO at our hospital were included in the study. The technical success rate, clinical success rate, time to recurrent biliary obstruction (TRBO) and overall survival (OS) from the first placement, factors affecting TRBO and OS, and early complications within 30 days after the procedure were evaluated retrospectively. (3) **Results:** The technical and clinical success rates were 100% and 96.6%, respectively, with a TRBO of 121 days [95% confidence interval: 82–231] and an OS of 194 days [95% confidence interval: 113–305] after the first placement. Early complications occurred in nine patients (15.3%), including five cases of cholangitis, three cases of pancreatitis, and one case of cholecystitis. (4) **Conclusions:** The PSIS method for UMHBO is safe and useful with high technical and clinical success rates.

## 1. Introduction

Although surgery is the only curative treatment for malignant hilar bile duct obstruction, up to 80% of patients are also diagnosed with unresectable malignant hilar biliary obstruction (UMHBO) [1,2]. Patients with UMHBO are typically treated with chemotherapy or palliative therapy. Chemotherapy prolongs survival, but jaundice and cholangitis should be well controlled with regular and continuous chemotherapy [3,4]. Bile stasis frequently occurs in UMHBO and leads to cholangitis, sepsis, decreased hepatic reserve, difficulty in continuing chemotherapy, and, eventually, a poor prognosis. It deteriorates the quality of life and survival even in patients receiving the best supportive care [5,6]. Good bile drainage is crucial in the treatment of patients with UMHBO.

For biliary drainage, two methods are used: endoscopic and percutaneous transhepatic biliary drainage. Endoscopic drainage is preferred to percutaneous transhepatic biliary drainage because it is more physiologic, less invasive and associated with a lower incidence of adverse events and shorter hospital stay [7,8]. Although endoscopic retrograde cholangiopancreatography is a widely standardized treatment procedure for obstructive jaundice [9], it can be technically challenging in some cases, with varying results in UMHBO among reports [10,11]. Endoscopic drainage in UMHBO is occasionally associated with serious complications, and percutaneous transhepatic biliary drainage should be considered as an alternative [10,12,13,14,15].

**Figure 1:**
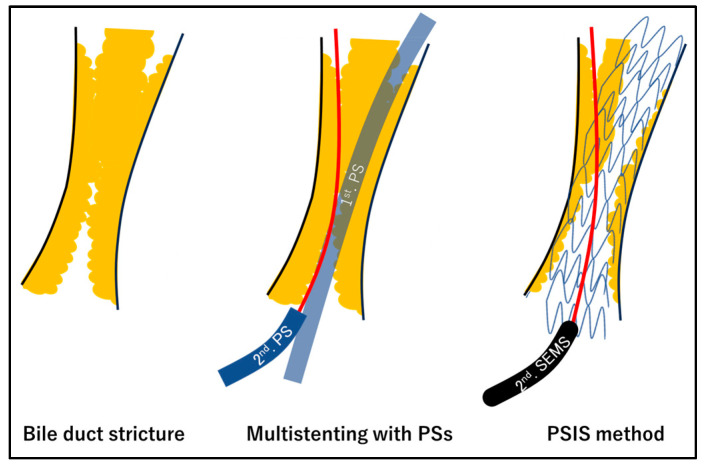
In the multistenting with PSs, once the first stent is placed, the second stent must pass through a narrower space than the first stent. Therefore, the second stent may have difficulty passing through the stricture. In the PSIS method, the stricture is dilated by the first SEMS. The second stent passes inside the first stent, allowing it to easily pass through the stricture.

Endoscopic biliary drainage is mainly performed with endoscopic bile duct stenting. However, the details of the procedure, including the drainage extent, stent type, and implantation method, are widely debated. Requisites for stenting include reliability, safety, short operation time, no decrease in quality of life, stent patency, and prolonged survival. Bilateral lobe drainage with the partial stent-in-stent (PSIS) method using a self-expandable metal stent (SEMS) is effective [1,16,17]. However, this method is often associated with high procedural difficulty [18]. In addition to the PSIS method, the side-by-side (SBS) method, which uses multiple SEMSs or plastic stents (PSs), is used for bilateral lobe drainage. The SBS method is considered less technically challenging than the PSIS method, and reintervention in the event of stent occlusion is relatively easy [19,20,21]. However, the SBS method, wherein stents are placed side-by-side in the bile duct, requires a larger space as the number of stents increases, and, in some cases, it may be more difficult to place stents using this method than with PSIS. For example, if the PS is placed using the SBS method, the smallest diameter of PS used is 7Fr. When multiple PSs are placed using the SBS method, space is required in the bile duct for the PSs. Therefore, there is considerable resistance when passing through the stricture, which may make placement challenging. Therefore, a high degree of difficulty is expected, especially when more than three PSs are to be placed (Figure 1).

**Figure 2:**
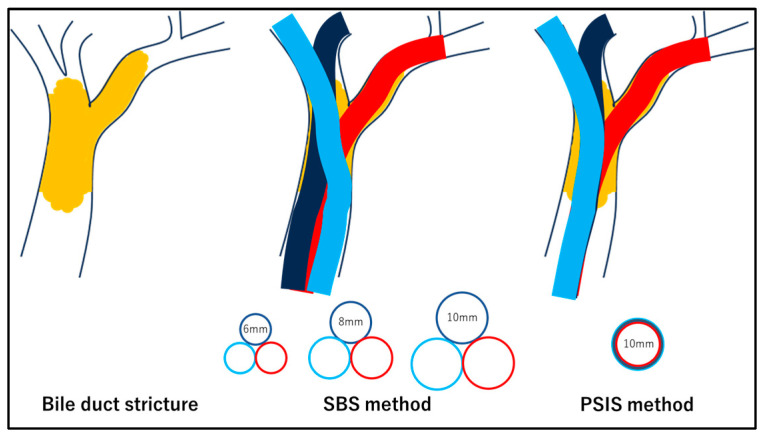
In the SBS method, stents are aligned side-by-side in the bile duct; therefore, the more stents there are, the more space is needed. Therefore, a high number of stents results in a state of common bile duct overexpansion. In the PSIS method, the second and subsequent stents are placed inside the first stent; thus, the common bile duct is not over-expanded even if the number of stents is increased, and a large number of stents can be placed.

**Figure 3:**
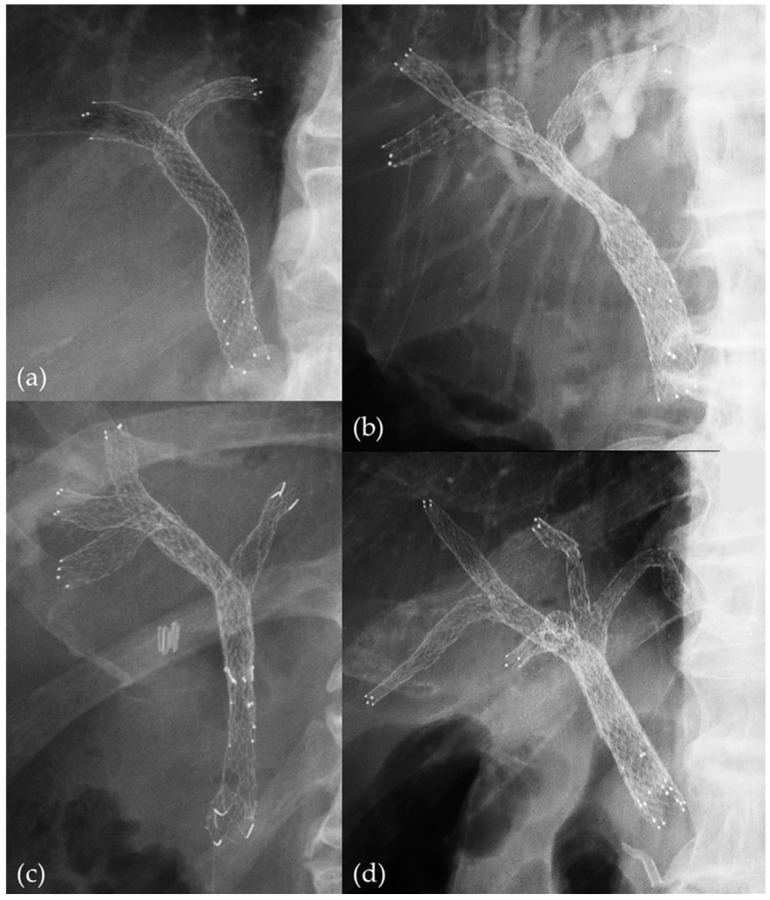
The PSIS method performed at our hospital: (**a**) Two SEMSs placed in a single procedure. (**b**) Three SEMSs placed in a single procedure. (**c**) Four SEMSs placed in a single procedure. (**d**) Five SEMSs placed in a single procedure.

When a SEMS is placed using the SBS method, it is likely to pass through the stricture more easily than PS owing to the thinner delivery systems of SEMSs. However, a single SEMS has a minimum diameter of 6 mm on expansion post placement, creating a risk of complications owing to common bile duct overexpansion, especially when more than three SEMSs are placed (Figure 2). In contrast, the PSIS method allows the first SEMS to pass through the stricture as easily as in the SBS method, and the second SEMS passes inside the expanded first SEMS; thus, there is no difficulty in passing through the stricture. In addition, the PSIS method does not require dilation of the common bile duct, allowing for the placement of larger-diameter SEMSs, and the drainage is more physiologic in nature (Figure 1 and Figure 2) [20,21]. The PSIS method allows the placement of SEMSs in a large number of bile ducts, and we have experience in placing 2–5 SEMSs in a single procedure (Figure 3). 

Nonetheless, there are two main points of difficulty in the PSIS method: the insertion of a guide wire into the target contralateral bile duct through the mesh gap from inside the first placed SEMS, and the insertion of the delivery system for the second SEMS through the mesh gap using the guide wire placed in the target bile duct and the deployment of the SEMS in the correct position. For this reason, the PSIS procedure is also often more technically challenging for reintervention at the time of stent occlusion. However, stenting for segmental cholangitis caused by a new obstruction of a bile duct may be easier to perform than reintervention using the SBS method. This is because the already placed SEMS can be used to approach the obstructed bile duct, to which a new SEMS can be added. Furthermore, newer devices are expected to improve the technical success rate and may become more common in the future [22,23,24,25].

Consequently, this retrospective study aimed to evaluate the usefulness and safety of the PSIS method for treating UMHBO at our institution.

## 2. Materials and Methods

This study was conducted in accordance with the Declaration of Helsinki. The study protocol was approved by the Ethics Committee of the Tottori University Hospital (approval no. 1508A024; 25 March 2022). We enrolled 59 patients who had undergone PSIS for UMHBO at our hospital from March 2017 to June 2021. In all cases, malignancy was diagnosed based on histopathological findings. Informed consent was obtained from all participants.

TJF-Q290V or JF-260V (Olympus Optical Co., Ltd., Tokyo, Japan) was used for the procedure. SEMSs were selected at the operator’s discretion from five types: Zilver 635^®^ Stent (Cook Medical Japan G.K., Tokyo, Japan), BileRush stent (Piolax, Inc., Kanagawa, Japan), Zeo stent V (ZEON Medical Inc., Tokyo, Japan), Niti-S Biliary Stent (Century Medical, Inc., Tokyo, Japan), and YABUSAME (Kaneka Medix Corp., Osaka, Japan). After bile duct intubation, a guidewire was placed in each bile duct targeted for stenting, and the first SEMS was placed along one guidewire. Subsequently, a guidewire was inserted into the contralateral bile duct through the mesh gap from inside the already placed SEMS using the other guidewire as a landmark, and a second SEMS was placed using this guidewire. The procedure described above was repeated for the placement of the third and subsequent SEMS.

The primary endpoints were the time to recurrent biliary obstruction (TRBO) and overall survival (OS) from first stenting. The secondary endpoints were the technical success rate, clinical success rate, factors affecting TRBO, factors affecting OS, and early complications within 30 days of the procedure.

**Figure 4:**
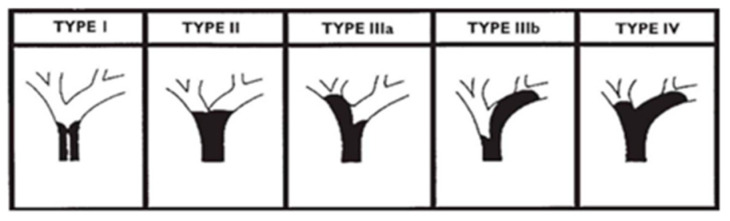
Bismuth classification. Type I: Tumor has not invaded the confluence of the right and left hepatic ducts. Type II: Tumor has invaded the confluence of the right and left hepatic ducts. Type IIIa: Tumor has invaded the secondary branch of the right intrahepatic bile duct. Type IIIb: Tumor has invaded the secondary branch of the left intrahepatic bile duct. Type IV: Tumor has invaded the secondary branches of the left and right intrahepatic bile ducts.

Technical success was defined as two or more SEMSs placed into each dilated bile duct beyond the stenosis and drainage confirmed on fluoroscopy. Clinical success was defined as reduction in the T-bil level to ≤50% within 1 week or ≤75% within 4 weeks after stenting. Early complications included procedure-related complications occurring within 30 days after the procedure. TRBO was calculated from the date of SEMS placement to the date of reintervention, the patient’s death, or the last follow-up visit. Obstruction of the hilar bile duct was classified into types I–IV based on the Bismuth classification. In this study, types IIIa and IIIb were grouped together as type III (Figure 4).

Kaplan–Meier and log-rank tests were used to test TRBO and OS. Median values were used for between-group comparisons using the Kaplan–Meier method. Univariate analysis was performed using the Cox proportional hazards model for each background factor, and multivariate analysis was performed for parameters with *p*-values < 0.05. A *p*-value < 0.05 was considered to indicate statistical significance. Statistical analyses were performed using EZR ver. 1.61 (Saitama Medical Center, Jichi Medical University, Saitama, Japan). EZR is a statistical software that extends the functionality of R (R Foundation for Statistical Computing, Vienna, Austria) and R Commander [26].

## 3. Results

### 3.1. Baseline Characteristics of the Patients

Table 1 shows the baseline patient characteristics. We enrolled 59 patients, including 39 (66%) men and 20 (34%) women, with a median age of 76 (68–81) years. The diseases included hilar cholangiocarcinoma (n = 17 [29%]), distal cholangiocarcinoma (n = 4 [7%]), gallbladder cancer (n = 14 [23%]), pancreatic cancer (n = 7 [12%]), intrahepatic bile duct cancer (n = 4 [7%]), hepatocellular carcinoma (n = 2 [3%]), and others (n = 11 [19%]). The Bismuth classification was as follows: type I, 3 (5%) cases; type II, 14 (23%) cases; type III, 17 (29%) cases; and type IV, 25 (42%) cases. Thirty-seven (63%) patients received chemotherapy after PSIS, and thirty-five (59%) patients received ursodeoxycholic acid.

### 3.2. Outcomes

The number of SEMSs was two in 34 (57.6%) patients, three in 22 (37.3%) patients, four in 2 (3%) patients, and five in 1 (1.7%) patient. The median TRBO from the initial stenting was 121 days (95% confidence interval: 82–231; Figure 5), and OS from the initial stenting was 194 days (95% confidence interval: 113–305; Figure 6). The technical success rate was 100% (59/59), and clinical success rate was 96.6% (57/59). The median total procedure time was 76 (59–113) min (Table 2).

### 3.3. Complications

Early complications occurred in nine (15.3%) patients, including five cases of cholangitis, three cases of pancreatitis, and one case of cholecystitis. All cases were mild and improved with conservative treatment alone.

### 3.4. Factors Influencing TRBO

**Figure 7:**
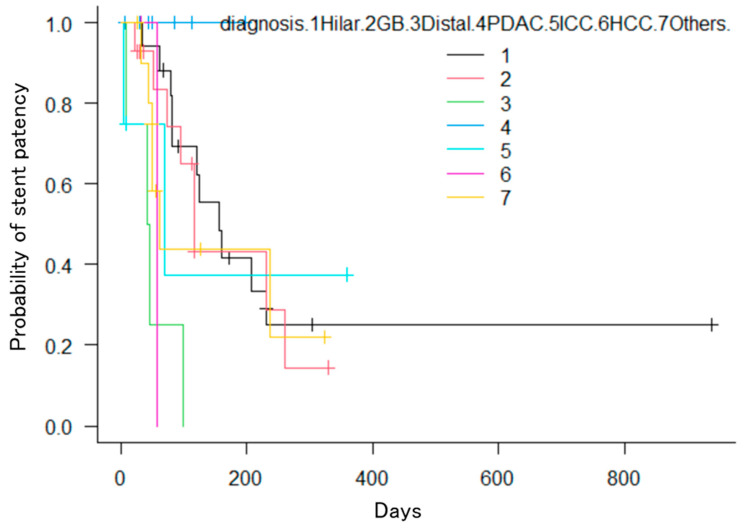
Kaplan–Meier estimation of the cumulative overall stent patency period. The perihilar cholangiocarcinoma group showed significant differences compared to the other groups (*p* = 0.016).

In the Kaplan–Meier method, patients with hilar bile duct cancer had significantly longer TRBO compared to those with distal bile duct cancer, gallbladder cancer, pancreatic cancer, intrahepatic bile duct cancer, hepatocellular carcinoma, or other cancers (*p* = 0.016; Figure 7). The number of stents (two vs. three or more, *p* = 0.81), chemotherapy (yes vs. no, *p* = 0.68), ursodeoxycholic acid (yes vs. no, *p* = 0.54), Bismuth classification (I vs. II vs. III vs. IV, *p* = 0.23), or drainage area (one lobe vs. both lobes, *p* = 0.65) showed no significant differences. Furthermore, univariate analysis using the Cox proportional hazards model showed no significant differences in either case.

### 3.5. Factors Influencing OS

**Figure 8:**
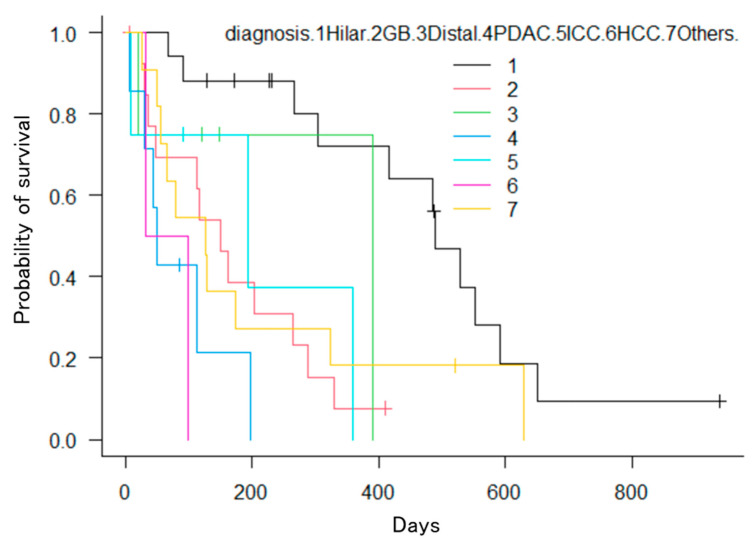
Kaplan–Meier estimation of the cumulative overall survival time. The perihilar cholangiocarcinoma group showed significant differences from the other groups (*p* = 0.0005).

**Figure 9:**
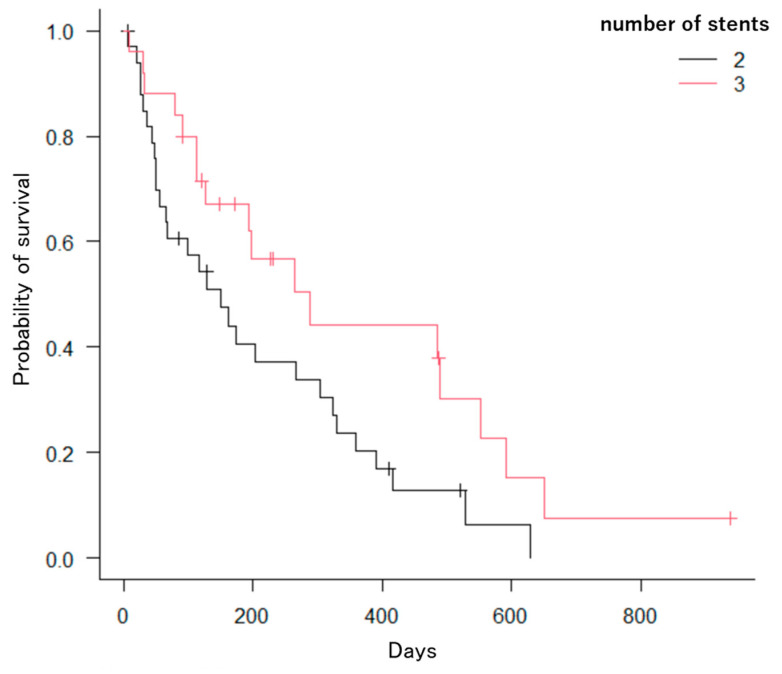
Kaplan–Meier estimation of the cumulative overall survival time (2: two, 3: more than three). Patients with more than three stents showed significant differences from those with two stents (*p* = 0.039).

**Figure 10:**
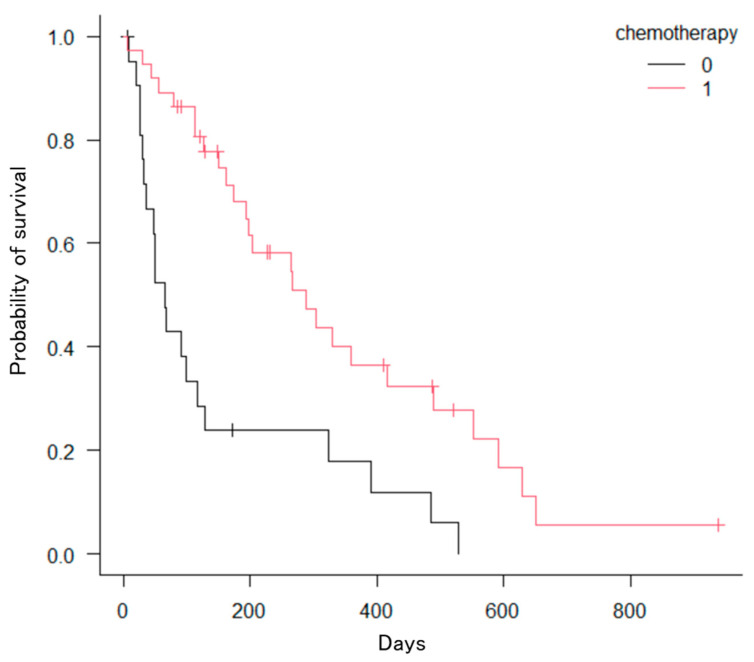
Kaplan–Meier estimation of the cumulative overall survival time. Patients treated with chemotherapy showed significant differences from those not treated with chemotherapy (*p* = 0.0002).

**Figure 11:**
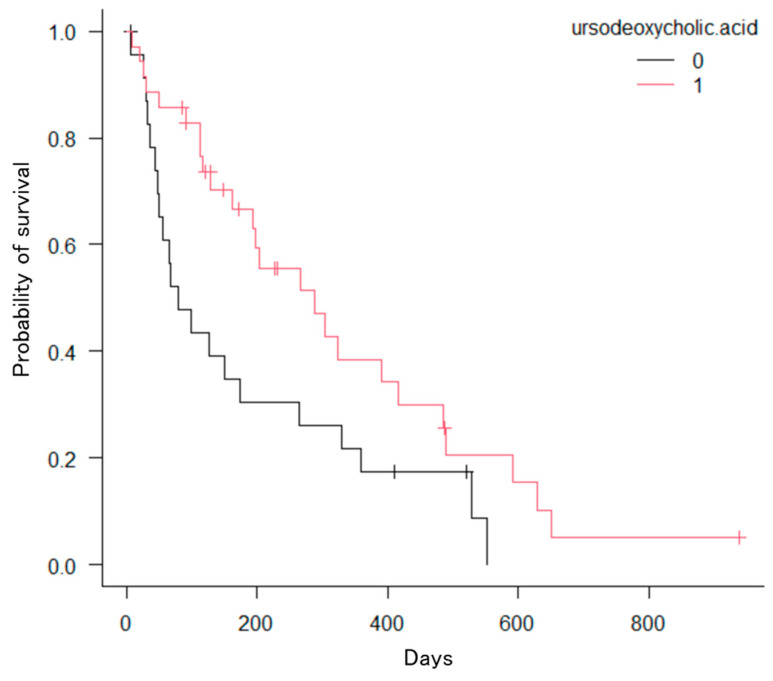
Kaplan–Meier estimation of the cumulative overall survival time. Patients who received ursodeoxycholic acid showed significant differences from those who did not (*p* = 0.027).

**Figure 12:**
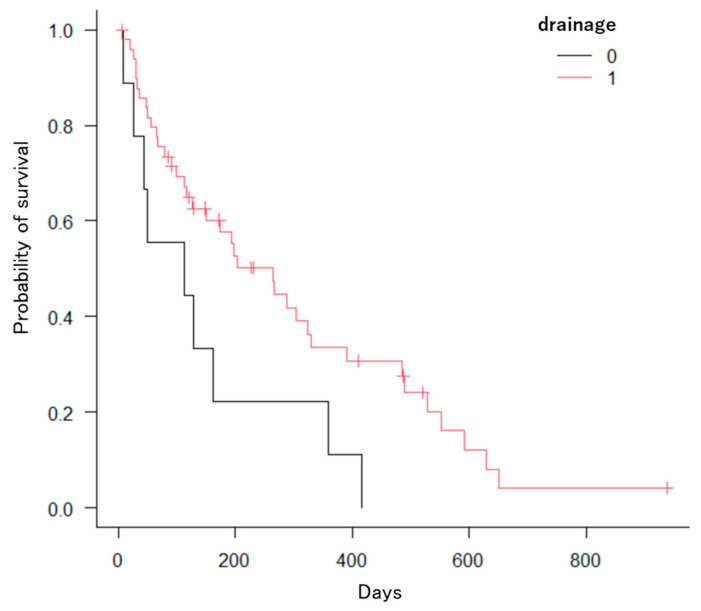
Kaplan–Meier estimation of the cumulative overall survival time (0: unilateral, 1: bilateral). Patients who underwent bilateral drainage showed significant differences from those who underwent unilateral drainage (*p* = 0.033).

**Figure 13:**
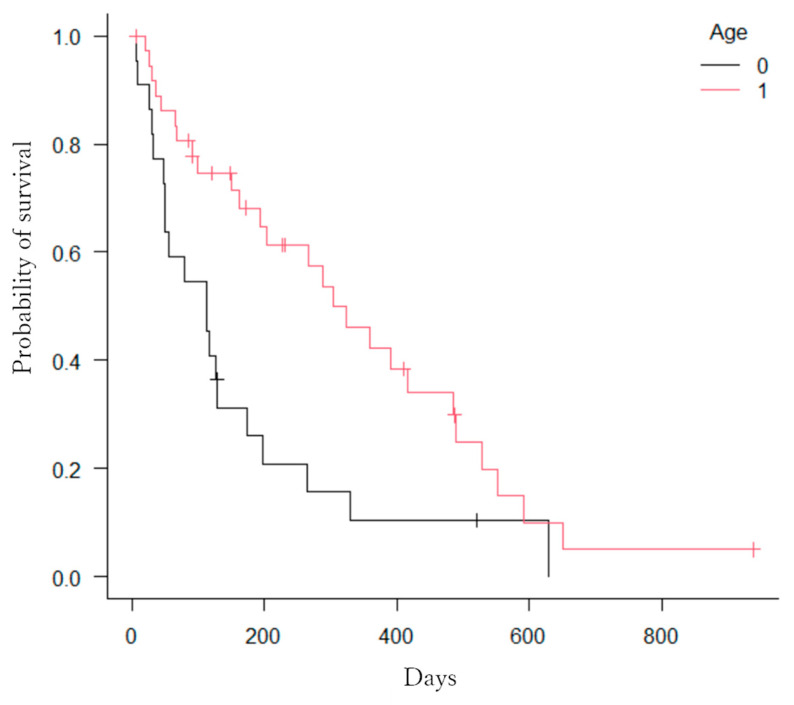
Kaplan–Meier estimation of cumulative overall survival time (0: years < 70, 1: 70 ≤ years). Statistically significant difference was observed between patients over 70 years old and those under 70 years old (*p* = 0.006).

The Kaplan–Meier method revealed significant differences in the number of factors. First, patients with hilar bile duct cancer had a significantly longer OS compared to those with distal bile duct cancer, gallbladder cancer, pancreatic cancer, intrahepatic bile duct cancer, hepatocellular carcinoma, or other cancers (*p* = 0.0005; Figure 8). Second, patients with three or more stents had significantly longer OS compared to those with two stents (*p* = 0.039; Figure 9). Third, patients who received chemotherapy had a significantly longer OS compared to those who did not (*p* = 0.0002; Figure 10). Fourth, patients who took oral ursodeoxycholic acid had a significantly longer OS compared to those who did not (*p* = 0.027; Figure 11). Fifth, patients who underwent bilateral drainage had longer OS compared to those who underwent unilateral drainage (*p* = 0.033; Figure 12). Sixth, patients aged over 70 years had a longer OS than patients aged under 70 years (*p* = 0.006; Figure 13). However, the Bismuth classification showed no significant differences (I vs. II vs. III vs. IV, *p* = 0.36). Univariate analysis with the Cox proportional hazards model showed similar results.

In a multivariate analysis of six parameters (hilar cholangiocarcinoma, three or more stents, chemotherapy, ursodeoxycholic acid, bilateral drainage, age), only chemotherapy was considered an independent factor for OS prolongation (Table 3).

## 4. Discussion

Malignant hilar biliary stricture has a poor prognosis, and endoscopic drainage is commonly performed for UMHBO [1,2,7,8]. UMHBO should be managed simultaneously with obstructive jaundice using chemotherapy, and improved drainage techniques are crucial for improving the prognosis of biliary tract cancer [3,4,5,6]. There is considerable controversy regarding drainage methods.

The extent of biliary drainage has long been debated between unilateral and bilateral [16,27,28,29,30]. Several meta-analyses have shown that bilateral lobe drainage with SEMS is associated with a lower incidence of stent dysfunction than unilateral drainage in patients with UMHBO [27,31]; however, various biases exist, including the stent type, stent placement approach, and disease type. A recent meta-analysis of hilar cholangiocarcinoma revealed no significant difference in technical (*p* = 0.52) or clinical success (*p* = 0.80) between unilateral and bilateral drainage with SEMS [32].

Unilateral stent insertion is sufficient to relieve jaundice associated with UMHBO because drainage of only 25–30% of the liver can normalize T-bil levels [33]. Another meta-analysis of endoscopic unilateral and bilateral SEMS placement for MHBO showed that the technical success rate was significantly higher in the unilateral than in the bilateral group [34]. However, a successfully drained liver volume is associated with functional success. Aiming for drainage of at least 50% of the liver volume using MRCP or CT reduces the risk of cholangitis and improves drainage efficacy and survival [35]. Bilateral stenting is essential to achieve drainage of >50% of the liver volume [19]. Recent improvements in stents have facilitated bilateral SEMS placement [22,23,24,25].

Whether PSs or SEMSs should be used for drainage is debatable [17]. In general, SEMS is recommended for patients with hilar cholangiocarcinoma whose survival is expected to be longer than 3 months, whereas PS is recommended for temporary drainage in patients with cholangitis whose treatment plan is undecided [36]. However, this consensus is based on evidence comparing SEMSs and PSs alone. Although SEMS has longer patency in distal cholangiocarcinoma [37,38], many recent studies have reported the superiority of SEMS for UMHBO in terms of stent patency, stent-related complications, and clinical success rate [36,39]. Although bilateral placement of SEMS is often technically difficult, the technical success rate is as high as 90–100% [16,29,30].

SEMSs can be placed in the hilar region with two methods: SBS and PSIS. Recent meta-analyses have reported longer patency with the PSIS method, and the usefulness of SEMS placement with the PSIS method has increasingly been reported [1]. The disadvantage of the PSIS method is often the high procedural difficulty of the first placement and reintervention; however, new devices are expected to improve the technical success rate [22,23,24,25].

The PSIS method using SEMS is technically difficult; therefore, recent developments in devices, such as thinner delivery systems and advances in guidewires and endoscopes, have had a significant impact. Further developments are expected to ease the difficulty of multi-stenting, which is considered difficult, and thereby improve the prognosis of biliary tract cancer in terms of drainage. In the present study, the PSIS method for UMHBO had a 100% technical success rate and a 96.6% clinical success rate. It was useful as a procedure that can be performed safely without major complications.

In the present study, disease was the only factor affecting TRBO after the first SEMS placement with the PSIS method, which showed a significant difference. Disease, three or more stents, chemotherapy, ursodeoxycholic acid, and bilateral drainage were also significant factors affecting OS after the first placement. Chemotherapy was found to significantly prolong OS in a prior study [3], consistent with our findings. Bilateral drainage did not significantly prolong TRBO but did significantly prolong OS, indicating its superiority over unilateral drainage, as previously reported.

Furthermore, the present study showed better OS with three or more stents than with two stents. Most patients with three or more stents had three-zone drainage, with drainage of the anterior and posterior areas of the right lobe in addition to both lobes, suggesting that conventional drainage of both lobes and drainage of the three areas from the first placement may improve the final prognosis. Therefore, further validation is required. On the other hand, the Inside PS method, wherein a plastic stent is placed in the bile duct above the duodenal papilla, has recently been reported to have a longer patency period [6]. This method has relatively low procedural difficulty and has the potential to become common in the future. However, the Inside PS method places the stent side-by-side in the common bile duct similar to the SBS method. Therefore, the problems of stricture passage and common bile duct overexpansion are similar between the two methods, and the number of stents that can be placed is limited. In fact, reports of the Inside PS method indicate that the number of stents placed is usually two or less; therefore, it may not be the optimal method for cases requiring three-area drainage. In contrast, our findings suggest that the PSIS method is a good alternative for cases requiring the drainage of three or more areas and, at present, may be superior to the Inside PS method for drainage of three or more areas. For bilateral drainage, the PSIS and Inside PS methods should be compared in the future. As the validation progresses, it may be possible to clarify indications for specific methods; for example, the Inside PS method could be used for bilateral drainage and the PSIS method could be used for the drainage of three or more areas.

In addition, ursodeoxycholic acid significantly prolonged OS in this study; however, the usefulness of ursodeoxycholic acid has seldom been reported.

This single-center, retrospective, observational study has several limitations. First, the number of cases and diversity were small, and the possibility of selection and information biases existed. Second, the operator skills were not uniform. Furthermore, multiple types of SEMSs were used, and no fixed criteria for selection were applied. In addition, some devices were different from the latest ones, which may have caused differences in the methods and difficulties in the procedures. Finally, although the median observation period was 148 days, the follow-up was short in many cases, and long-term follow-up may not have been sufficient. The present study showed that OS was significantly better in patients with three or more SEMSs than in those with two SEMSs; however, some cases with three SEMSs did not have three drainage areas. Although this study suggests the usefulness of three drainage areas, a larger prospective study is required for a more accurate evaluation.

## 5. Conclusions

The PSIS method with SEMSs for UMHBO is a safe procedure with a high success rate. In this study, the placement of three or more SEMSs significantly prolonged the patients’ OS compared to the placement of two SEMSs. This suggests that three-area drainage may be superior to bilateral drainage. Furthermore, three stents must be placed for three-area drainage, and the PSIS method may be appropriate for the placement of three or more stents. Furthermore, for the placement of three or more SEMSs, the PSIS method has advantages even in reintervention. Although the PSIS method has been cited as challenging owing to its high degree of procedural difficulty, recent advances in devices have made it easier, and it is likely to become more common in the future. The Inside PS method has recently been reported as a less difficult drainage method with a longer patency period, but there are few cases of more than three stents being placed using this method. Further comparisons with the PSIS method may clarify the indications for each method.

## Figures and Tables

**Figure 5:**
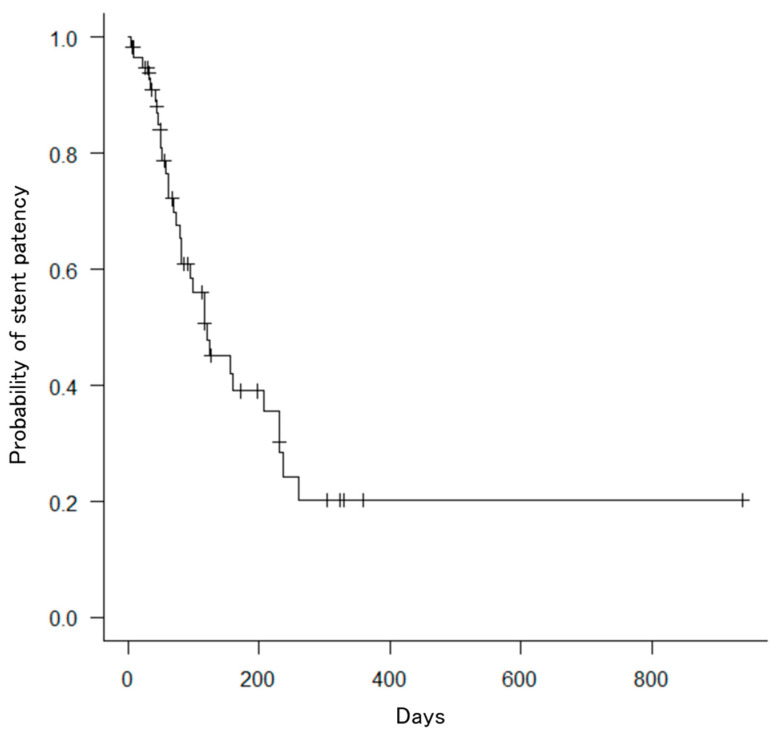
Kaplan–Meier curve for the cumulative stent patency of bilateral metallic stents. The median survival time was 121 days.

**Figure 6:**
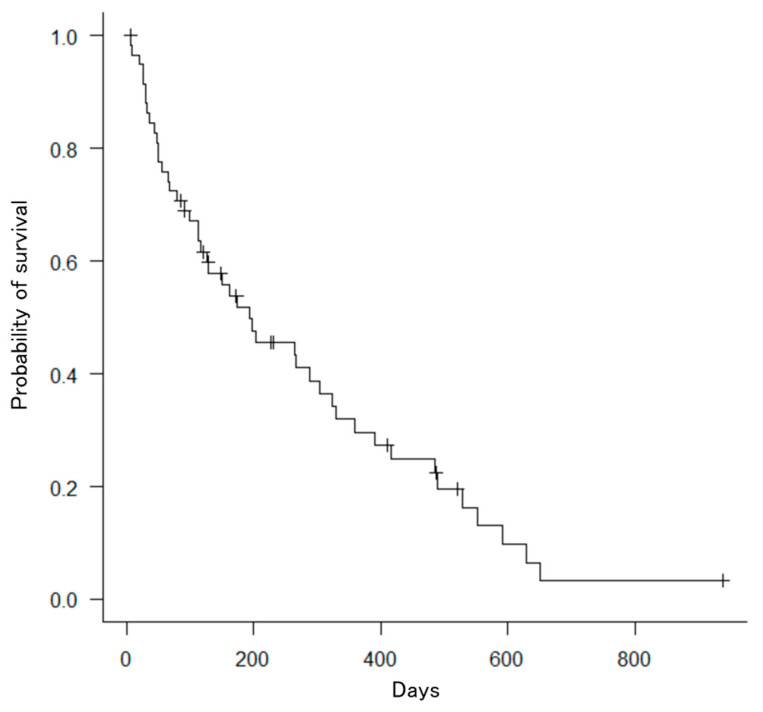
Kaplan–Meier curve for overall survival after placement of bilateral metallic stents. The median survival time was 194 days.

**Table 1:** Patient characteristics.

Characteristics	n = 59
Sex (male/female)	39/20
Age (years), median (IQR)	76 (68–81)
Diagnosis	
Hilar cholangiocarcinoma	17 (29%)
Distal cholangiocarcinoma	4 (7%)
Gallbladder cancer	14 (23%)
PDAC	7 (12%)
ICC	4 (7%)
HCC	2 (3%)
Other tumors	11 (19%)
Bismuth classification	
I	3 (5%)
II	14 (23%)
III	17 (29%)
IV	25 (42%)
Chemotherapy	37 (63%)
Ursodeoxycholic acid	35 (59%)

Other tumors included three cases of colon cancer, three cases of ovarian cancer, three cases of gastric cancer, two cases of lung cancer, one case of gastrointestinal stromal tumor, and one case of uterine cervical cancer. IQR, interquartile range; PDAC, pancreatic ductal adenocarcinoma; ICC, intrahepatic cholangiocarcinoma; HCC, hepatocellular carcinoma.

**Table 2:** Outcomes.

Outcomes	n = 59
Number of stents	
Two	34 (57.6%)
Three	22 (37.3%)
Four	2 (3%)
Five	1 (1.7%)
Procedure time (min), median (IQR)	76 (59–113)
Technical success	59 (100%)
Clinical success	57 (96.6%)
Early complications	9 (15.3%)
Cholangitis/pancreatitis/cholecystitis	5/3/1
TRBO (days), median (95% CI)	121 (82–231)
OS (days), median (95% CI)	194 (113–305)

IQR, interquartile range; TRBO, time to recurrent biliary obstruction; CI, confidence interval; OS, overall survival.

**Table 3:** Independent factors associated with survival after partial stent-in-stent with a self-expandable metal stent for unresectable malignant hilar biliary obstruction in the Cox regression analysis.

Variables	Univariate Analysis			Multivariate Analysis		
	HR	95% CI	*p*-Value	HR	95% CI	*p*-Value
Age (70 ≤ years)	0.445	0.244–0.811	0.008	0.454	0.209–0.987	0.046
Female	1.850	0.974–3.516	0.060			
Chemotherapy	0.331	0.179–0.612	0.0004	0.270	0.136–0.534	0.0001
Hilar cholangiocarcinoma	1.149	1.025–1.289	0.017	1.039	0.889–1.213	0.630
Three or more stents	0.525	0.282–0.977	0.042	1.270	0.587–2.745	0.543
Bilateral drainage	0.452	0.214–0.956	0.037	0.456	0.203–1.024	0.057
Ursodeoxycholic acid	0.514	0.282–0.929	0.030	0.553	0.289–1.060	0.074

HR, hazard ratio; CI, confidence interval.

## Data Availability

The datasets generated during and/or analyzed during the current study are available from the corresponding author upon reasonable request.
